# Editorial: Systems biocatalysis for bioprocess design

**DOI:** 10.3389/fbioe.2022.1010174

**Published:** 2022-11-09

**Authors:** Roland Wohlgemuth, Jennifer Littlechild, Byung-Gee Kim

**Affiliations:** ^1^ Institute of Molecular and Industrial Biotechnology, Lodz University of Technology, Lodz, Poland; ^2^ Swiss Coordination Committee for Biotechnology, Zurich, Switzerland; ^3^ Henry Wellcome Building for Biocatalysis, Biosciences, University of Exeter, Exeter, United Kingdom; ^4^ School of Chemical and Biological Engineering, Institute of Molecular Biology and Genetics, Seoul National University, Seoul, South Korea; ^5^ Bio-MAX/N-Bio Institute & Institute for Sustainable Development (ISD), Seoul National University, Seoul, South Korea

**Keywords:** biocatalysis, biotransformation, biosynthesis, biocatalytic process design, biocatalytic reaction engineering

Biocatalysis is continuing to be of key importance and relevance for the functioning of natural as well as anthropogenic bioprocesses on planet earth. There is a growing awareness directed to the application of bioprocesses in creating and preserving value, by avoiding loss of value and regenerating new value from waste products. This approach is intended to address climate change and sustainability, which are the great challenges of the 21st century. The favorable features of biocatalytic processes, such as safety, health, environmental sustainability, high selectivity, and resource efficiency, are attracting increasing attention for both synthetic and degradative bioprocesses at a small to large industrial scale.

Bioprocess design and value creation in a sustainable bioeconomy can benefit from an integrated view, which includes the molecular and engineering aspects of the optimal biocatalytic transformation route. The thermodynamics of the biocatalytic transformations and the kinetic characteristics and properties of the involved biocatalysts are key determinants for the resource and energy efficiency of the bioprocesses. This is true whether the transformation route is to be designed for the synthesis of a desired target or from a starting material oriented perspective, and in addition whether it is for a single given target or is oriented towards a diversity of products. The search, design, and optimization of the biocatalysts can benefit from a large diversity of different approaches and methodologies. This can start from biocatalyst identification in biosynthetic pathways already existing in nature, from *de novo* design and evolution of engineered biocatalysts or from a combination of these approaches. The selection of the most suitable starting materials for the bioprocess can be guided by renewable and bio-based raw materials used in natural biosynthetic pathways, by bio-privileged compounds, or by economic factors such as availability, cost, and source of the starting materials. The range of the desired target products can benefit from the creation of better bio-products containing new functionalities to achieve an improved performance.

Systems approaches provide valuable tools and methodologies for designing new bioprocesses, by offering a thorough insight into the required system components and interactions. Integration of insights into the kinetics and thermodynamics of individual reaction steps within biocatalytic reaction pathways that exist in nature can help new efficient industrial processes to be developed. This process identifies the natural starting materials and their intermediates to obtain the desired products and requires a multidisciplinary approach involving biology, chemistry, and engineering for the bioprocess design. The systems biocatalysis approach includes whole-cell *in vivo* analysis of biocatalytic systems, retrosynthetic analysis, discovery of new biocatalytic functions, development and production of highly active and stable biocatalysts, reaction engineering of individual and multiple biocatalytic reaction steps, downstream processing, and product recovery. Based on the rapidly growing knowledge regarding the structure, function, mechanism, and application of enzymes, the development of biocatalytic reaction pathways using a systems biocatalysis approach can guide the design of resource efficient and sustainable bioprocesses towards the manufacturing of valuable target products. The tasks involved are the discovery of new biocatalysts, the optimization of their function, mutual compatibility and regulation, and the construction of a continuous flux within a synthetic pathway to obtain an optimal efficiency.

This special issue contains a collection of reviews and original research papers that build on the development of this key area of research which strives to achieve a sustainable future worldwide. The contributions range from the development of stable enzyme fusions for improved catalytic performance, and the discovery of new enzyme catalysts and their optimization of substrate specificity using directed and rational mutagenesis, to their use for the biosynthesis of a range of important small metabolites and metabolite-like molecules. The papers within this special topic as outlined below describe a range of new biosynthetic opportunities using either a whole-cell *in vivo* approach or an *in vitro* cell-free approach, using purified enzyme catalysts. In addition, the importance of modelling the biocatalytic process is introduced. This can often be a vital task to carry out at an early stage of development to evaluate the feasibility of the proposed reactions and to allow any subsequent redesign for scale-up to industrial scale to be fully optimized.

Reductive aminases are highly attractive enantioselective biocatalysts in the reductive coupling of carbonyl compounds with primary and secondary amines for synthesizing chiral amines. The broad substrate scope and other favorable properties of two new reductive aminases have been explored and characterized for the direct biocatalytic synthesis of primary and secondary amines, and control of enantioselectivity in rasagiline synthesis has been demonstrated using enzyme engineering by rational design (Zhang et al.). Site-directed mutagenesis has been used in engineering cytochrome P450 monooxygenase CYP153A33 from *Marinobacter aquaeolei* for obtaining increased hydroxylation at the ω-position of 1-dodecanol (Park et al.). The new enzyme variant CYP153A33 P136A has also been investigated with respect to ω-specific hydroxylation of C6 to C16 1-alkanols. A review on methanol dehydrogenases, which catalyze the conversion of methanol to formaldehyde and play a key role in natural or artificial methanol utilization pathways, describes the discovery, properties, and applications of new and newly engineered methanol dehydrogenases (Le et al.). A mini-review on the design of fusion enzymes, which focusses on fusion oxidoreductases with their reported linkers and applications in both aqueous and non-aqueous media, discusses key efficiency and feasibility limitations of aqueous biocatalysis, use of non-aqueous media, and the potential of fusion oxidoreductases in organic media (Ma et al.). A single whole-cell system, which co-expresses the required enzymes in a reaction cascade and the controlled expression level of each enzyme, is demonstrated. This efficiently catalyzes the conversion of ethyl 3-oxo-4-(2,4,5-trifluorophenyl) butanoate to a sitagliptin intermediate, using an esterase, and a transaminase from *Roseomonas deserti*, with benzylamine as amino donor, whereby aldehyde reductase from *Synechocystis* sp. and formate dehydrogenase from *Pseudomonas* sp. were used to resolve the inhibitory effect of benzaldehyde (Khobragade et al.). Despite the natural occurrence of metabolites, new efficient methods for their preparation in adequate amounts are clearly needed to study their biological activities, as described for the enzymatic synthesis of the regiospecific enantiomers 3′-hydroxy-(*S*)-equol, 5-hydroxy-(*S*)-equol, and 6-hydroxy-(*S*)-equol from daidzein and genistein, using daidzein*-(S)*-equol–converting reductases and 4-hydroxyphenylacetate 3-monooxygenase (Song et al.). As α-amino ester hydrolases are of much interest in cephalexin manufacturing, rapid evaluation is desirable regarding the influence of the reactor type and configuration, and on the conversion of the reactant, the cephalexin yield, and the volumetric productivity, taking into account process characteristics such as enzyme deactivation and substrate inhibition (Lagerman et al.). Utilizing inexpensive resources and hetero- or phototrophic metabolic modules for a sustainable supply of the necessary cofactors or co-substrates is attractive for redox-intensive whole-cell biocatalysis. This aims to increase regeneration and supply of redox equivalents, block competing fluxes, and increase the required metabolite availability for the desired biosynthetic routes (Theodosiou et al.). A systems biocatalysis approach and modular cell-free biocatalytic systems offer new opportunities for reducing the complexity of synthesizing metabolites using biological whole-cell approaches or by classical chemical synthesis (Wohlgemuth and Littlechild). The identification of the proteins involved in inulin utilization by *Faecalibacterium prausnitzii* and a multi-omic study have provided molecular insights with respect to inulin metabolism in *F. prausnitzii* and its connection to improving human intestinal health (Park et al.).

Together, the papers included in this special issue on systems biocatalysis offer important contributions to our drive towards the development of new sustainable industrial bioprocesses. They also provide an overview of the challenges that are involved and how these can be overcome using a systems biocatalysis approach.

